# Supplementary material for: The Construction and Exploration of a Comprehensive MicroRNA Centered Regulatory Network in Foxtail Millet (Setaria italica L.)
Source: Front Plant Sci. 2022 May 6;13:848474. doi: 10.3389/fpls.2022.848474 (PMC9121102; doi:10.3389/fpls.2022.848474)
Supplement: Supplementary file 2 [file Data_Sheet_2.zip › Sit-miR396a_Seita.1G316200.1_3186_TPlot.pdf]

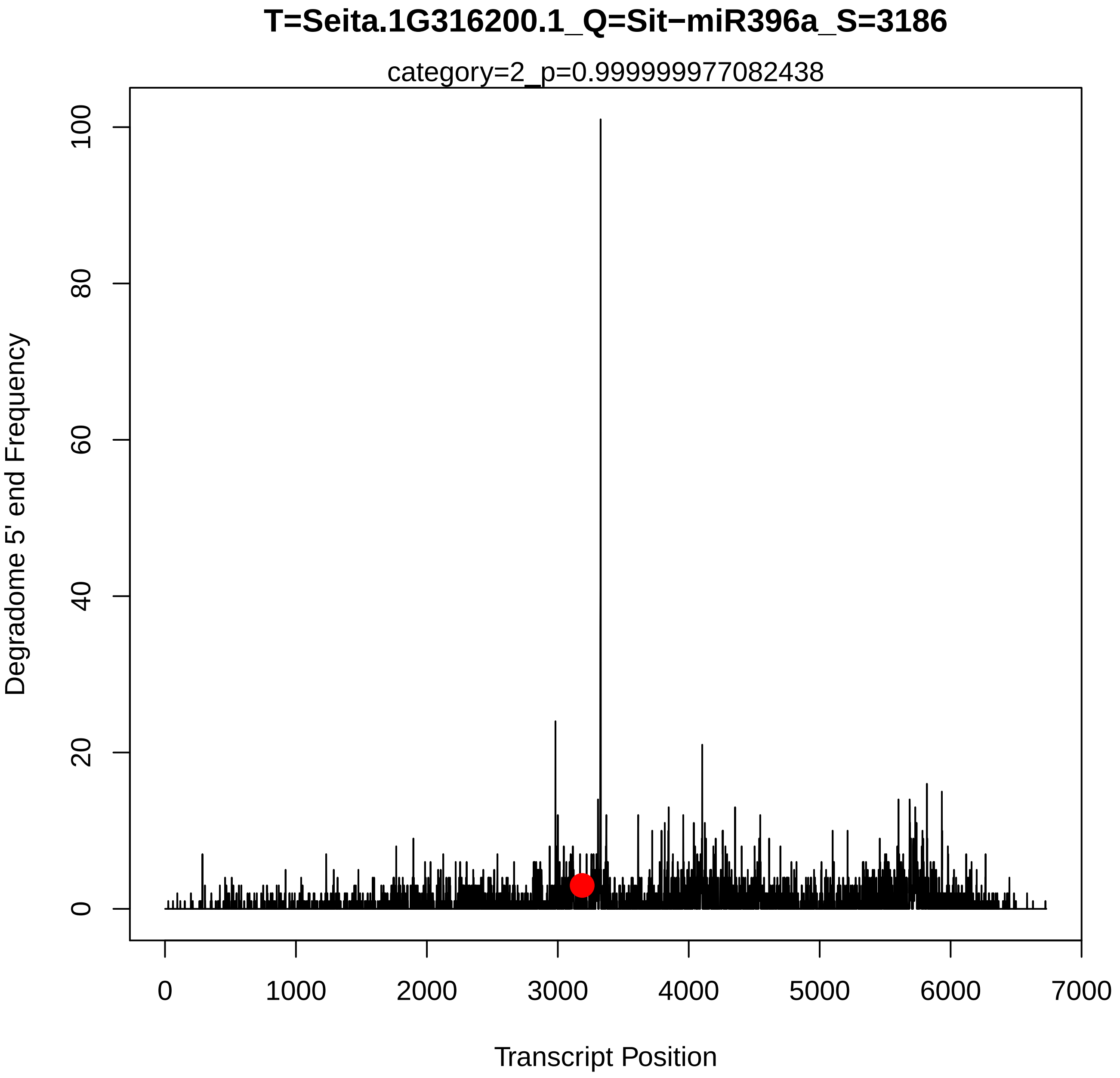
T=Seita.1G316200.1_Q=Sit−miR396a_S=3186
category=2_p=0.999999977082438
Degradome 5' end Frequency
0
20
40
60
80
100
0
1000
2000
3000
4000
5000
6000
7000
Transcript Position
